# Droplet microfluidics for single-cell studies: a frontier in ecological understanding of microbiomes

**DOI:** 10.1093/femsre/fuaf032

**Published:** 2025-07-23

**Authors:** Wannes Nauwynck, Karoline Faust, Nico Boon

**Affiliations:** Ghent University, Center for Microbial Ecology and Technology (CMET), Department of Biotechnology, Frieda Saeysstraat 1, 9052, Gent, Belgium; KULeuven, Laboratory of Molecular Bacteriology (Rega Institute), Department of Microbiology, Immunology and Transplantation, Herestraat 49, 3000, Leuven, Belgium; KULeuven, Laboratory of Molecular Bacteriology (Rega Institute), Department of Microbiology, Immunology and Transplantation, Herestraat 49, 3000, Leuven, Belgium; Ghent University, Center for Microbial Ecology and Technology (CMET), Department of Biotechnology, Frieda Saeysstraat 1, 9052, Gent, Belgium

**Keywords:** single-cell technologies, droplet microfluidics, microbial ecology, functionality, microbiology, double emulsion, bacterial interactions, cultivation

## Abstract

Recent advances in single-cell technologies have profoundly impacted our understanding of microbial communities—shedding light on cell-to-cell variability in gene expression, regulatory dynamics, and metabolic potential. These approaches have shown that microbial populations are more heterogeneous and functionally complex than previously thought. However, direct probing of single-cell physiology—arguably more ecologically relevant by focusing on functional traits such as growth, metabolic activity, and enzymatic activity—remains underexplored. Droplet microfluidics provides a practical and high-throughput approach to address this gap, allowing functional characterization of individual microbial cells within complex communities and offering new opportunities to study ecological processes at high resolution. In this review, we look at the state of droplet microfluidics for single-cell microbial ecology. We revisit the fundamentals of microbial droplet workflows, we overview the current capabilities of droplet microfluidics that exist for microbial ecology and we look at the phenomena these workflows have uncovered and understanding they have generated. Finally, we integrate these capabilities to envision future droplet workflows that could enhance our understanding of single-cell physiology and discuss the fundamental limitations that go together with the droplet format.

## Teaser

Review and synthesis of recent droplet microfluidics approaches for probing single-cell microbial physiology, highlighting current ecological insights and future potential for functional trait analysis and interaction screening in complex communities.

Acronyms: FACS: Fluorescence-activated cell sorting
FADS: Fluorescence-activated droplet sorting

## Introduction

Over the past few decades, microbiome research has relied heavily on bulk sequencing technologies, which provide population-level insights in microbial composition, functional profiles and gene expression. These approaches have revealed that most microbiomes host a rich array of species with diverse metabolic capabilities (Thompson et al. 2017). However, despite the wealth of genomic and expression data, a thorough ecological understanding and predictive theoretical framework of microbiomes is lacking (Prosser 2015, 2020). This lack of understanding can—in part—be explained by the fact that microbiome composition, functionality, and dynamics ultimately emerge from the physiological states (phenotypes) and activities (functionalities) of individual microbial cells. Gene content and gene expression, while insightful, generate an overload of data, from which the individual phenotype or functionality of single cells are not extractable (Buccitelli and Selbach 2020). Probing physiology and functionality of interest directly offers more readily interpretable information. It is therefore crucial to measure and understand single-cell physiology to grasp and predict the functioning of microbial communities.

To better appreciate the importance of physiological measurements at the single-cell level, it is important to recognize that even genetically identical microbial cells can exhibit different phenotypes. This means that microbial cells from the same strain can exhibit different physiologies through stochastic events or through exposure to different environments (Ackermann 2015, Heyse et al. 2019, García-Timermans, Rubbens et al. 2020). Examples of this can be found in the form of bet-hedging by *Escherichia coli* cells exposed to antibiotics in which stochasticity in gene expression leads to a subset of the population turning dormant, allowing them to survive antibiotic exposure (Rotem et al. 2010). Other examples include heterogeneous toxin production and even metabolic specialization within species (Ceuppens et al. 2013, A. Z. Rosenthal et al. 2018). It is clear that understanding such phenotypic variations is crucial, as microbial phenotypes such as dormancy, stress responses, and metabolic activity shape how cells interact with each other and their environment, ultimately influencing broader community functions. Despite these known physiological phenomena, our understanding remains incomplete, particularly when applied to complex microbial communities (Ackermann 2015, García-Timermans, Rubbens et al. 2020).

A key example of the lacking understanding of this single-cell physiology in microbiomes is the persistent nature of ‘the great plate count anomaly’. Why can we cultivate one bacterium from a microbial community and not another? This question is a fundamental one in microbiology and microbial ecology but there is still no clear agreement on whether most ‘uncultivable’ bacteria cells are dead, dormant, if they are missing an obligate mutualistic partner or whether their specific nutritional needs are unmet. With the advent of better sequencing technologies, observations of the phenomenon have become increasingly more precise but the dominant mechanisms remain elusive (Staley and Konopka 1985, Ward et al. 1990, Epstein 2013, Lewis et al. 2021, D. Wu et al. 2025). It is clear that to study and understand such a fundamental phenomenon, methodologies are needed that allow studying, measuring and experimenting of microbial physiology and functionality at the single-cell level where these phenomena occur.

Currently physiological questions are answered by isolating the desired microbial members from the community. Besides cultivability issues, cultivation-based approaches are time and labor-intensive, limiting the number of cultures that can be screened to a few thousand (Wittebolle et al. 2009). This fundamental limitation is why cultivation-based approaches operate on the species-level and mostly disregard strain-level diversity, as the sheer number of strains generally associated with microbiomes is too vast to test comprehensively (Viver et al. 2024). Additionally, the isolated members undergo domestication, a process that alters the phenotypical state of the microbial cell found in the community to one that is more readily propagated in the lab (Yu et al. 2016). It is clear that cultivation-based approaches paint a biased picture of a microbiome, and different approaches are needed.

Over the past decades droplet microfluidics has matured, and a suite of tools is now available for fine-tuned control over droplet formation, merging and splitting, which enables complex manipulations within a high-throughput framework (Moragues et al. 2023). Because of this, droplet microfluidics is a promising candidate technology for dealing with the vast diversity encountered in microbial communities at a single-cell level. In this review, we take a closer look at current state-of-the-art applications of droplet microfluidics for the study of single-cell physiology and we critically assess its potential to further our ecological understanding of microbial communities. To this end, we revisit the fundamentals of microbial droplet workflows, provide an overview of the current capabilities of droplet microfluidics for microbial ecology and highlight key phenomena these workflows have already uncovered. In a following section, we integrate these current capabilities into future droplet workflows, while highlighting the understanding they could bring of single-cell physiology. Finally, we conclude by underlining the fundamental limitations of the droplet format and outlining the challenges that must be addressed to realize its potential in microbial research.

## The basics of droplet microfluidics

The application of droplet microfluidics in microbial ecology takes many forms: from the detection of extracellular metabolites and enzymes to screening interactions in microbial communities. However, all possible workflows are governed by the same set of rules and experimental constraints, which are important to grasp to critically assess the strengths and weaknesses of droplet techniques. We shortly introduce them in the following section.

### Droplet generation and encapsulation of microbes

In general, every droplet workflow consists of three steps: droplet generation, droplet manipulation, and droplet analysis. During droplet generation a uniform population of microbe-containing droplets is made. This is followed by droplet manipulation (optional), which entails a series of microfluidic steps that manipulate the initial population, much like pipetting steps in standard lab workflows. Each unique step requires a unique chip design and so far, designs for droplet washing, droplet splitting, droplet merging, droplet mixing and droplet dilution have been developed. These techniques have been recently reviewed (Moragues et al. 2023). The final step is droplet analysis, potentially combined with droplet sorting. Here, droplet data is gathered and droplets of interest can be scaled up to microliter-scale experiments through sorting, allowing linking up with existing lab techniques which enables easy processing. The droplet workflows discussed in this review vary in complexity: some make use of many different droplet manipulation techniques, some workflows do not use any droplet manipulation at all (i.e. only make use of droplet generation and droplet analysis).

**Figure 1.**
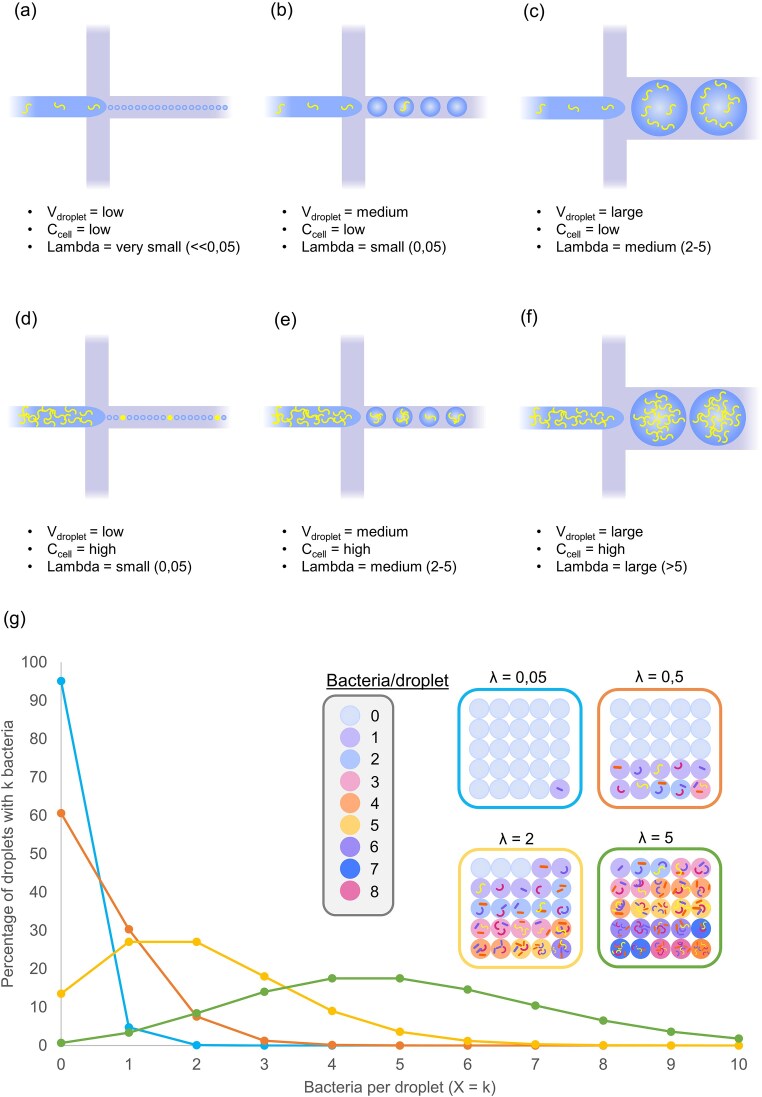
Visualization of droplet generation, the parameter lambda and Poisson distribution. (a)-(f) Schematics of flow-focusing droplet generation chips with varying cell concentrations (C_cell_) and droplet volumes (V_drop_). The schematics show droplets being generated, of which the microbial load is described by the Poisson distribution and the parameter lambda. (g) Poisson distributions of different lambda values. The x-axis represents the number of microbial cells per droplet (k) and the y-axis represents the fraction of droplets that contain k microbial cells. There are 4 different λ-values with each their Poisson distribution plotted and a schematic of a sample of the droplet population. The Poisson distributions from most left-skewed to most right-skewed are λ = 0.05 (blue), λ = 0.5 (orange), λ = 2 (yellow) and λ = 5 (green). E.g. for lambda = 0.05, there are 95% empty droplets, 5% of droplets contain 1 microbial cell per droplet, more than 1 microbial cells per droplet is a negligible fraction.

The first and most important step in any droplet workflow is the generation of uniform microdroplets, which sets the experiment's foundation. In this step, a microbe-containing aqueous sample and an immiscible oil phase are introduced into a microfluidic chip through separate microchannels. As these two liquids flow towards a junction, the aqueous phase is segmented into discrete droplets by the surrounding oil (Fig. 1 a-f). During this process, shear forces overcome interfacial tension and pinch off droplets at a constant rate (Moragues et al. 2023). Since microbial cells are floating throughout this aqueous sample, their encapsulation happens at random—each droplet captures microbial cells only if cells are present in the break-off point at the moment of droplet formation (Fig. 1a-f).

The chance that microbial cells are present in an encapsulated droplet is determined by the volume of the droplet and the microbial concentration of the sample in the following way:


\begin{eqnarray*}
\lambda = \,\,{C_{\textit{cells}}}*{V_{\textit{droplet}}}
\end{eqnarray*}

with C_cells_ being the cell concentration (cells/mL) of the cell suspension entering the chip and V_droplet_ being the volume of the droplets (pL). Thus for high cell concentrations (10^9^ cells/mL) and large droplet volumes (100 pL) this chance is high (λ = 100, Fig. 1f), since on average, a 100 pL defined volume of this sample will contain 100 microbial cells. On the contrary, for low concentrations (10^6^ cells/mL) and small droplet volumes (1 pL) this chance is low (λ = 0.001, Fig. 1a), since on average, a 1 pL defined volume of this sample will only contain 0.001 cell. This kind of stochastic behavior can be modelled with the Poisson distribution.

In the specific case of droplet microfluidics, the probability distribution that is modelled describes the initial droplet population; namely the percentage of droplets that contain zero microbial cells, one microbial cell, two microbial cells etc. at timepoint zero, just after droplet production (Fig. 1g).

The Poisson distribution can be described by following equation:


\begin{eqnarray*}
P\left( {X = x} \right) = \,\,\frac{{\left( {{e^{ - \lambda }}\,\,{\lambda ^x}\,\,} \right)}}{{x!}}
\end{eqnarray*}

with x = number of microbial cells per droplet and P(X = x) representing the probability a droplet is formed containing x microbial cells in strict terms, but in terms of initial droplet population it represents the percentage of droplets that contain x microbial cells. As is shown by the previous two equations, the distribution is completely determined by the droplet volume and the cell concentration, which means that the initial distribution of microbial cells in droplets can be set by controlling these two parameters. Cell concentration can be changed by diluting or concentrating the sample using standard pipetting techniques. Depending on the geometry and channel height of the chip, droplets with volumes ranging from femtoliters to nanoliters can be generated (Boedicker et al. 2008, Leman et al. 2015). To allow sufficient growth, most microbial workflows make use of droplets ranging from picoliter to nanoliter volumes (Terekhov et al. 2018, Kehe et al. 2019). The initial distribution of microbial cells in droplets has important consequences for experimental design. It locks in place the starting conditions of an experiment, i.e. the number of founder cells in a droplet (Fig. 1g). At low λ values, where single-cell droplets dominate, analysis can proceed under the assumption that each droplet contains only one cell, minimizing biases introduced by multiplets. For example, for λ = 0.05, of all the droplets that contain a cell, more than 95% are single-cell droplets, and less than 5% are droplets that started out with multiple cells (Fig. 1g). Therefore, since only a small fraction of non-single-cell droplets are present, analysis of these droplets happens under the assumption that all cell-containing droplets are single-cell droplets. However, as seen later in this review, experiments that investigate interactions between different microbial species sometimes employ higher λ values (Terekhov et al. 2018). At these higher concentrations, droplets contain a mix of single and multi-cell starting conditions, making it difficult to determine the exact founder cell composition without advanced experimental design (i.e. fluorescent labelling or sequencing) or direct observation methods (microscopy).

Because these droplet assays can take on a multitude of forms depending on inoculation strategies and measurement readouts (growth or no growth in droplets), we introduce a clear nomenclature to distinguish between them throughout this review (see Box 1).

Box 1. Clarification of droplet-based assay terminology used in this review.

Droplet-based microbiology workflows vary across three main parameters:

- Number of cells inoculated per droplet (single-cell (low lambda) vs. multi-cell (high lambda))
- Whether growth occurs before measurement (measurement vs. outgrowth measurement
- Number of genotypes per droplet (inoculation vs. co-inoculation)

To maintain clarity, we consistently define our terminology based on these combinations. We use inoculation to describe the starting composition of cells within droplets and measurement to describe the state of the population at the point of analysis.

**Inoculation terminology:**

- Single-cell inoculation: Droplets seeded with a single cell of a single genotype.
- Multi-cell inoculation: Droplets seeded with multiple cells of a single genotype.
- Co-inoculation: Droplets seeded with cells from multiple genotypes (one or more cells per genotype).

**Measurement terminology:**

- Single-cell measurement: Direct measurement of a single inoculated cell without growth.
- Multi-cell measurement: Direct measurement of multiple inoculated cells without growth.
- Outgrowth measurement: Measurement after allowing growth within droplets.
  Single-cell outgrowth measurement: From droplets seeded with a single cell.
  Multi-cell outgrowth measurement: From droplets seeded with multiple cells.

**Interaction assays:**

When droplets contain multiple genotypes, and growth occurs, we refer to these as interaction assays, as the readout reflects the outcome of co-cultivation. For clarity, we drop the explicit “co-inoculation” in these cases:

- Single-cell interaction assay: Typically starting from lambda ∼2–5 cells per droplet.
- Population-level interaction assay: Starting from lambda > 5 cells per droplet.

In practice, Poisson encapsulation leads to distributions of cell numbers and genotypes per droplet. Our terminology reflects the experimental design intent rather than exact per-droplet control.

**Figure B1:**
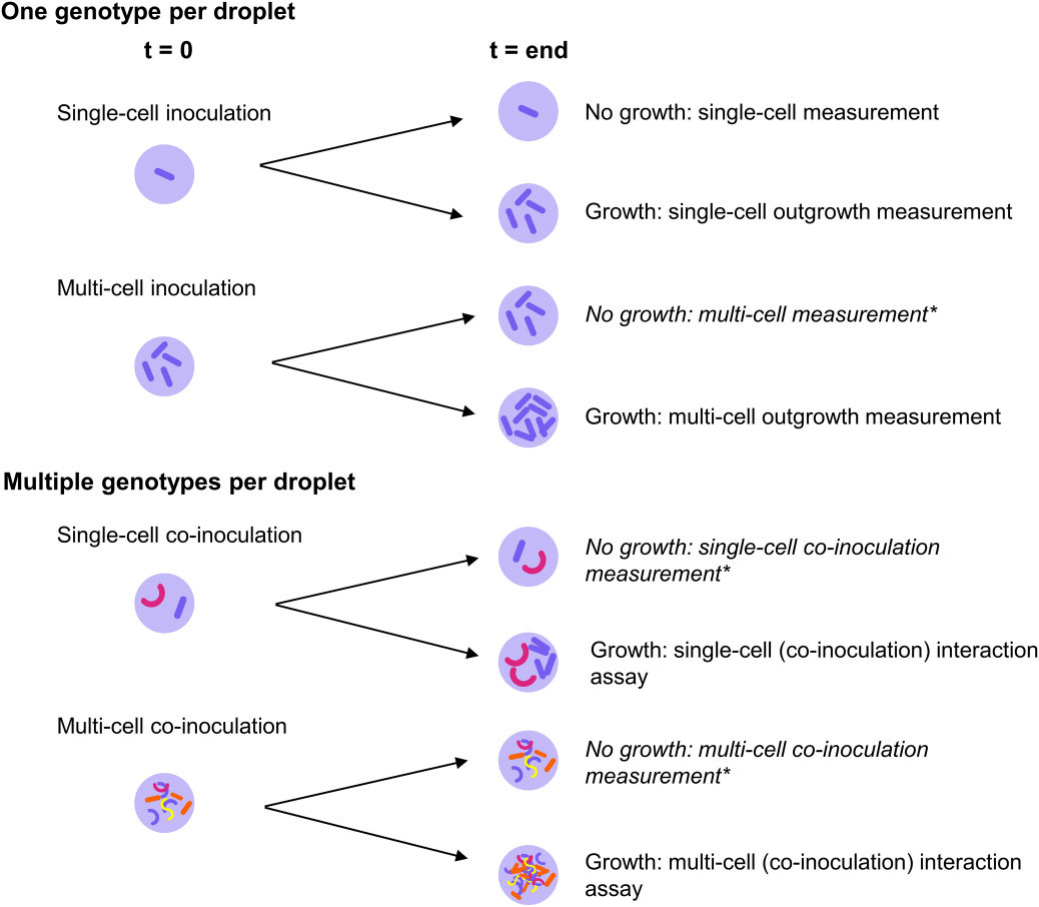
Overview of droplet assay terminology as used in this review. Schematic representation of the combinations of key parameters determining the terminology of dropletbased microbiology assays: (1) the number of cells per droplet (single-cell vs. multi-cell inoculation), (2) the number of genotypes per droplet (single genotype vs. co-inoculation of multiple genotypes), and (3) whether or not outgrowth occurs prior to measurement (direct measurement vs. outgrowth measurement).

This framework provides a terminology throughout the review and is visually summarized in Figure B1.

Schematic representation of the combinations of key parameters determining the terminology of droplet-based microbiology assays: (1) the number of cells per droplet (single-cell vs. multi-cell inoculation), (2) the number of genotypes per droplet (single genotype vs. co-inoculation of multiple genotypes), and (3) whether or not outgrowth occurs prior to measurement (direct measurement vs. outgrowth measurement). ‘*’ represents unused terminology in this review but that was included for the sake of completeness.

### Droplet analysis and sorting

After droplet generation, the emulsions are manipulated to obtain the desired experimental conditions. This goes from simple manipulations such as exposing the droplets to a certain temperature to grow the encapsulated microbes, to more complex manipulations, such as adding lysing agents or adding additional assay reagents to each droplet on microfluidic chips. A myriad of droplet manipulation techniques exist, but a detailed discussion of these is out of the scope of this review. For an overview we refer readers to Moragues et al. (2023). In the end, droplets are analyzed and data can be gathered about the microbial population in question.

Although the miniaturization into droplets offers a significant advantage in terms of throughput—microfluidic systems can generate up to 10 000 droplets per second, and a single milliliter of droplets can contain approximately 10⁸ picoliter-sized compartments (Moragues et al. 2023)—it also comes with trade-offs. Standard analytical techniques are incompatible with droplet-based workflows, as they often rely on macroscale bulk measurements rather than individual droplet interrogation. As such, detection methods must be adapted to operate at a droplet level, ensuring that signals can be reliably measured from such tiny volumes (Zhu and Fang 2013).

Most microbial droplet workflows make use of fluorescence-based methods. This is because fluorescence is sensitive enough to get quantitative signal from the picoliter droplet volumes and allows flexible assay development since many commercial options are available under the form of stains, fluorescent proteins or other fluorescent molecules. On top of this, widespread fluorescence-based techniques such as microscopy and flow cytometry (and fluorescence-activated cell sorting (FACS)) are compatible with the ultrahigh-throughput nature of droplet workflows, enabling analysis of up to 1000s of events per second (Brower et al. 2020). Other analytical techniques such as Raman spectroscopy, mass spectrometry and nuclear magnetic resonance spectroscopy have also been adapted for droplet analysis but have lower throughput, are technically complex and only available to specialized labs. For an overview of non-fluorescent droplet analysis techniques we refer readers to Liu and Zhu (2020) and Zhu and Fang (2013).

Sorting of droplets is a highly desirable capability during analysis since it allows isolating a desired droplet and linking it to standard lab techniques such as nucleic acid extraction, nucleic acid amplification, sequencing and standard cultivation techniques. Two options exist. Firstly, droplets can be sorted on a custom chip, requiring a retrofitted fluorescence microscope to perform. This fluorescence-activated droplet sorting (FADS) technique is highly custom in nature, and is therefore generally only seen in specialized microfluidics labs (Panwar et al. 2023, Sukovich et al. 2017). A second technology through which droplets can be sorted is FACS, a commercially mature sorting technology that offers extremely high throughput (up to 10 000 events/s) and advanced capabilities compared to FADS with many more colors and automation capabilities (Brower et al. 2020, Zhuang et al. 2024).

It is important to note that droplets are uniform and indistinguishable from each other. To track a droplet over time, it needs to be assigned a droplet identity. This can be done by labelling a droplet with a unique combination of fluorophores (Kulesa et al. 2018), by fixing the droplet position in a 2D-array (Kehe et al. 2019), or by fixing the order of the droplets in a narrow channel (Cao et al. 2017). This way one droplet can be addressed several times since the droplets are identified by their unique fluorophore combination, coordinates or rank. However, there are drawbacks to these approaches. When using color codes to label droplets, a portion of the available fluorescence channels must be dedicated to identification, reducing the number of channels available for experimental measurements, limiting experimental flexibility. When droplets are organized spatially, such as in a 2D-array, throughput is reduced because expansion into the third dimension is not possible; and additionally, advanced droplet manipulations such as picoinjection and fusion become challenging, as current technologies do not reliably maintain droplets in fixed positions.

## Current capabilities for microbial ecology

Understanding microbial physiology at the single-cell level offers valuable insights for microbial ecology, as many ecological processes emerge from single-cell behaviors. This section explores current methods developed to investigate microbial physiology at the single-cell level, focusing on enzyme activity assays, metabolite measurements, and interaction-based assays. While current capabilities for functionality screens offer the flexibility needed for diverse assay designs in microbial ecology, it is important to note that they have so far been applied predominantly in the context of enzyme evolution, and have yet to see widespread application in microbiome screening efforts. Here, we highlight these methods to showcase their potential for advancing microbial ecology research.

### Functionality-based screens

#### Measuring enzyme activity

A powerful property of droplet microfluidic workflows is the linking of (secreted) phenotype to the genotype of the encapsulated microbes. These workflows can directly extract a microbial cell with a desired functionality from a complex microbial population or measure the state of the functionalities of a vast number of cells. Here we go over the most common methods to establish functionality (i.e. measuring metabolites and enzyme activity) in microdroplets.

**Figure 2.**
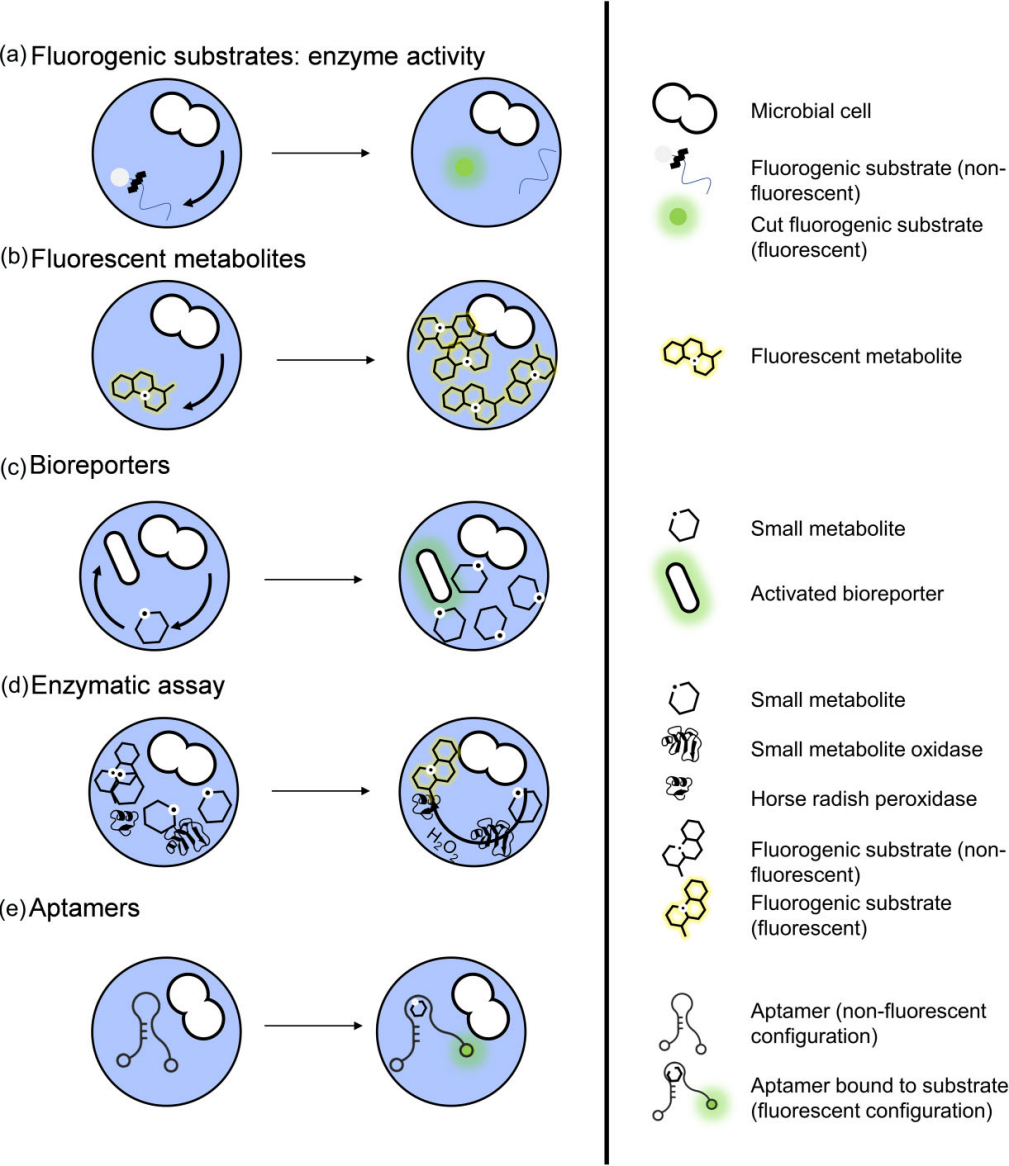
Fluorescent functionality screening methods in droplet microfluidics. Schematic overview of various approaches to assess cellular functions at the single-cell level (or of single-cell outgrowth) within droplets: (a) Fluorogenic substrates detect specific enzymatic activities by producing a fluorescent signal upon substrate conversion. (b) Fluorescent metabolites allow for direct detection of secreted or accumulated fluorescent products. (c) Bioreporters involve engineered cells that emit a fluorescence signal in response to a target metabolite. (d) Enzymatic assays couple cellular metabolite production to the production of a fluorescent signal through a double enzymatic reaction scheme. The metabolite of interest is oxidized, producing hydrogen peroxide. This hydrogen peroxide is then used by horseradish peroxidase to reduce a fluorogenic compound which in turn turns fluorescent. (e) Aptamer-based sensors use oligonucleotides that bind specific metabolites, inducing a conformational change that results in fluorescence.

To detect desired enzyme activities directly, fluorogenic substrates are most commonly used. These molecules are initially non-fluorescent, but are rendered fluorescent through the activity of the enzyme of interest (Fig. 2a). For the study of microbiome functions, a broad range of fluorogenic substrates have already been developed, allowing detection of oxidoreductases, glycosidases, esterases, lipases, peptidases, DNases, phosphatases and proteases (Manafi et al. 1991, Miller et al. 1998, Pala et al. 2020). These molecules have been extensively used in more traditional experimental settings and since they rely on fluorescence as a read-out, they are easily amenable for droplet workflows. The most notable example of this is the use of calcein violet-AM to detect esterase activity of the outgrowth of single cells in droplets of an oral microbial community (Terekhov et al. 2017, 2018). The authors assume active cells to contain working esterase, which allows distinguishing droplets containing active cells based on the amount of calcein violet-AM that is cleaved into its fluorescent counterpart. Another recent study uses a difluoro-4-methylumbelliferyl β-d-xylobiose-based droplet assay to screen for improved single-cell xylanase activity in *Komagataella pastoris* (Ma et al. 2019). A final example is the use of BODIPY-labeled casein to screen for improved protease activity using droplets. In this case, digestion of the casein by the protease releases the fluorescent BODIPY and increases fluorescent signal of the analyzed droplet (Holstein et al. 2021).

#### Measuring metabolite concentration

##### Fluorescent metabolites

Many approaches exist for measuring metabolite concentration through fluorescence. When the metabolite of interest is fluorescent, screening is straightforward, with metabolite titers inside the droplet being linearly correlated with its corresponding fluorescent signal (Fig. 2b). In this way, Chen et al. (2017) succeeded in extracting a riboflavin-overproducing strain that exhibited 4-fold higher secretion capabilities after screening droplets with single-cell outgrowth from a mutation library.

##### Bioreporters for metabolite detection

Advances in genetic engineering and synthetic biology have allowed the development of suites of bioreporter (i.e. cells that detect metabolites and transform metabolite concentration into a fluorescent signal) for the detection of a variety of small molecules (Y. Wu et al. 2020). Combining this approach with the high throughput of droplet workflows allows rapid screens of heterogeneous microbial populations (Fig. 2c). Bowman et al. (2021) demonstrate this by screening three mutagenized libraries to enrich for highly productive strains. The screening relied on single-cell outgrowth measurements with each library combined with its metabolite-specific bioreporter: *E. coli* for naringenin, *E. coli* for triacetic acid lactone, and *Saccharomyces cerevisiae* for L-DOPA. In similar fashion, Siedler et al. (2017) use p-coumaric acid-sensitive *E. coli* bioreporters in droplets to screen mutagenized libraries. Saleski et al. (2019) demonstrated that single-cell outgrowth screenings can be used to detect production of cross-fed metabolites by using an auxotrophic bioreporter (*E. coli)*, demonstrating its efficiency for both 2-ketoisovalerate and L-tryptophan. H. Lee et al. (2020) developed a droplet-based bioreporter assay to detect methane monooxygenase activity of outgrowth populations of single cells, based on the monooxygenases ability to convert benzene to phenol. This phenol is detected by a phenol-sensitive bioreporter. Reporter strains can also test broader ecologically relevant functionalities. An example of this can be seen in the 2018 paper of Mahler et al. where bioreporters are used to detect the production of antibiotics. They used red fluorescent strains of *Bacillus subtilis 3610* and *E. coli* ECJW992 to detect the effect of antibiotic production of the single-cell outgrowth from a soil microbiome—low fluorescence indicating that antibiotics are affecting the growth of the bioreporters.

##### Enzymatic assays for metabolite detection

More general methods for metabolite screening are promising developments in the field. In their 2014 paper, Wang et al. develop a fluorescent assay design rationale that enables the fluorescent detection of a wide variety of metabolites (B. L. Wang et al. 2014). The rationale relies on the enzymatic oxidation of a metabolite of interest, which generates hydrogen peroxide. The hydrogen peroxide is used by horseradish peroxidase, converting a fluorogenic compound (Amplex™ UltraRed) from a non-fluorescent to a fluorescent state (Fig. 2d). They designed assays for the detection of xylose and L-lactate in droplets. With it, they screen a mixed population of *S. cerevisiae* and extract rare xylose-overconsuming cells after single-cell outgrowth in droplets. Additionally, they enriched rare L-lactate-producing *E. coli* from a population of mainly D-lactate-producing *E. coli*. It has recently been applied to evolve enzymatic activity of alditol oxidase at ultrahigh throughput (R. G. Rosenthal et al. 2023). This method relies on oxidation of the metabolite of interest, so it can only be used for molecules that can be oxidized. Other limitations are the compatibility of the growth assay with the enzymatic buffer and the availability of commercially available metabolite oxidases.

##### Fluorescent aptamer metabolite detection

Another general method for fluorescent metabolite detection is the use of DNA or RNA aptamers. These oligonucleotides can specifically bind to their target metabolites with high affinity and selectivity. The structural flexibility of oligonucleotides enables the design of biosensors that can adopt a wide range of three-dimensional configurations to specifically bind any target molecule, making aptamers highly adaptable tools for biosensing applications. In their 2017 article, Abatemarco et al. introduce the RNA-APtamers-In-Droplets (RAPID) method as a novel high-throughput screening technique of single-cell outgrowth (Abatemarco et al. 2017). This innovative approach leverages the sensitivity and specificity of RNA aptamers, enabling the detection of secreted metabolites in microdroplets at ultrahigh throughput. By employing “Spinach” aptamers, which translate the concentration of a secreted target molecule into a fluorescent signal (Fig. 2e), RAPID allows for the rapid fluorescent screening and sorting of millions of droplets. The study demonstrates the practical application of RAPID by significantly enhancing the production of tyrosine and the secretion of recombinant proteins in *S. cerevisiae*, achieving up to 28-fold and 3-fold increases respectively. Another recent study demonstrates the use of tryptophan DNA aptamers in droplets for the enrichment of highly active TrpB proteins by single-cell screening (Scheele et al. 2024). However, this technology also faces several challenges: aptamer design remains largely empirical and labor-intensive; the aptamer folding conditions must be compatible with the requirements for cellular growth within droplets; and the aptamers themselves must be stable against potential degradation by nucleases (DeRosa et al. 2023).

##### Raman spectroscopy

An emerging technique for droplet analysis is Raman spectroscopy, which relies on inelastic scattering—a process where photons interact with molecules and undergo an energy shift that reflects the molecule's vibrational properties, which provides information about the molecular bonds present in the sample (K. S. Lee et al. 2021, 2024). In this way, Raman spectroscopy allows the fingerprinting and in the best cases, the quantification of different molecule groups by measuring the shift in wavelength of the light scattered by the sample in comparison with the incident light. This enables label-free droplet fingerprinting based on their chemical contents. The developments for microbial single-cell functionality screening are promising (García-Timermans, Props et al. 2020, García-Timermans, Rubbens et al. 2020, K. S. Lee et al. 2024) but key limitations remain: Raman signals are weak, spectra often overlap in complex samples, and as such, background can interfere with detection. Molecules that have characteristic Raman signatures can be identified, but quantification is generally difficult. An exception is isotope labeling, where spectral shifts enable quantification of isotope uptake (Chisanga et al. 2021), offering opportunities to study single-cell metabolic variation. Given these challenges, we foresee the best application scenarios for droplet-based Raman analysis in defined media, targeting specific, known compounds to facilitate interpretation and deconvolution.

### Interaction-based screens

Measuring microbial interactions is essential for understanding ecosystem dynamics and functions. Traditionally, batch experiments are used to compare growth of species A in monoculture with growth of species A in coculture with species B (Gause 1934). Such experiments are straightforward and widely used but struggle with higher-order interactions due to the impracticality of testing all possible species combinations. Other approaches focus on reconstructing the interaction network more efficiently, for example by using leave-one-out community experiments (n experiments for n microbial species) at steady-state to parameterize generalized Lotka-Volterra (gLV) models (Venturelli et al. 2018). These methods help infer pairwise interactions within a community context, but they quantify only ‘effective’ pairwise interactions, which represent the net effect between species pairs and can subsume underlying higher-order interactions. Furthermore, they require steady-state conditions and inherit the limitations of the gLV framework they use (Ansari et al. 2021): it does not describe higher-order interactions explicitly and assumes that interaction strengths are constant over time and scale linearly with species abundance. This excludes the modeling of nonlinear effects such as interaction saturation, which may be ecologically relevant, for instance in cross-feeding or resource competition scenarios. Other methods allow studying metabolite-mediated many-to-one interactions such as using semi-permeable membranes to separate a single species from a community and spent-medium assays (Heyse et al. 2019, Schmitz et al. 2024). These simplify interaction measurements but they introduce their own limitations: they capture only metabolite-mediated interactions, and make it hard to disentangle who in the community releases which metabolites. Finally, network inference is a popular exploratory data analysis technique that allows generating hypotheses about biotic interactions based on measurements of taxon abundances across samples. However, the false positive rate is high due to challenges such as confounding factors, data pre-processing issues, and the inability to infer causal relationships (Faust 2021).

Droplet microfluidics offers a promising route to overcome the throughput limitations inherent to classical interaction screens. By enabling massively parallel screening—on the order of hundreds of millions of microdroplets—it allows probing large numbers of species combinations and higher-order interactions. In this section, we explore droplet-based interaction screening methods that fall into two broad categories. The first derives interaction measurements from cultured isolates, where subcommunities are reconstituted from a pool of cultivated species by random combination. The second, which we refer to as native interaction profiling, captures interactions from microbial communities without prior cultivation. In this approach, individual cells are encapsulated directly after extraction from their native environment—such as soil, aquatic, or gut samples–preserving their composition, native phenotypic states and avoiding culture-induced biases. These droplet-based screens can therefore assess pairwise or more complex interactions in a context that is closely informed by their natural ecological setting. Both strategies use the throughput and miniaturization advantages of droplet microfluidics but differ in control, ecological relevance, and experimental design.

#### Interaction screening with cultured isolates

**Figure 3.**
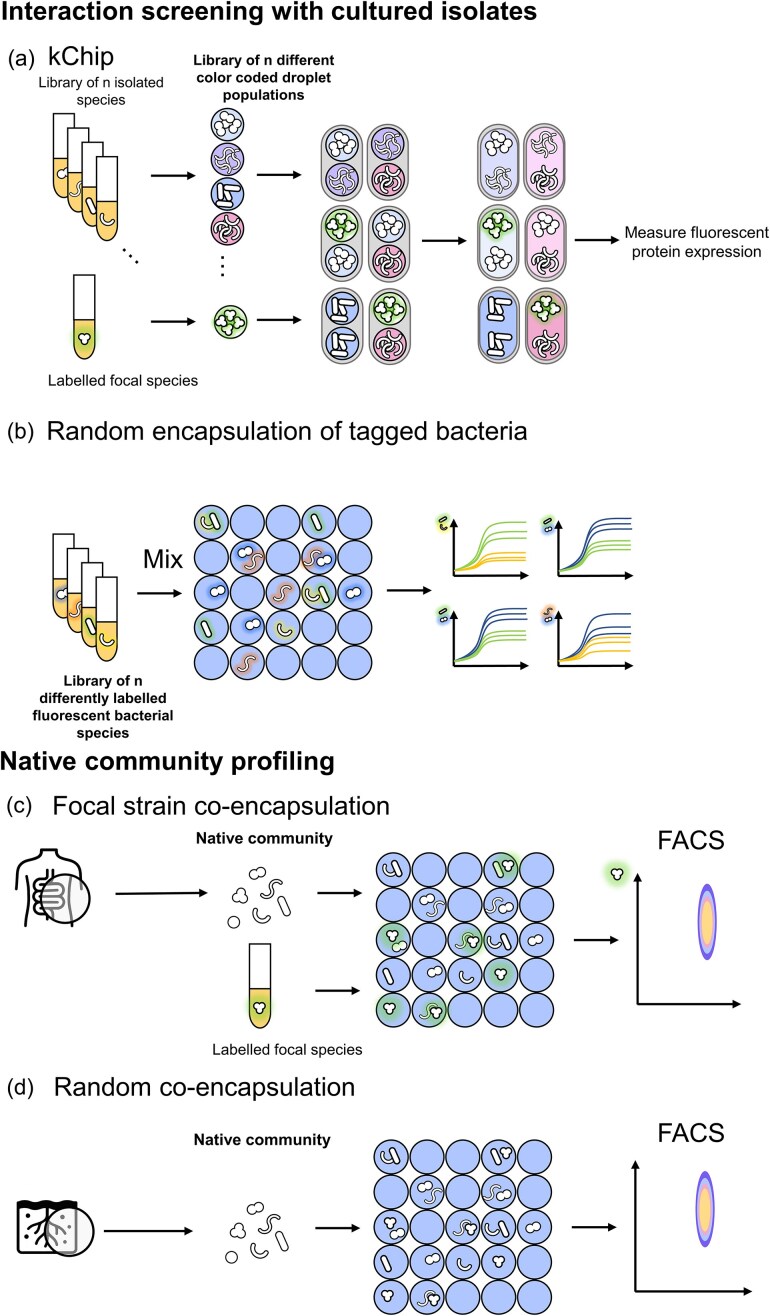
Overview of droplet-based interaction screening strategies. (a) kChip pairwise screening: A library of n microbial isolates is prepared, where in each of n experiments a different isolate is fluorescently tagged (e.g. with green fluorescent protein). The remaining isolates are identified by their droplet colour code. Multi-cell inoculated droplet populations for each isolate are generated, randomly paired and fused. After incubation, the growth of the tagged strain is quantified via fluorescent protein expression, serving as a proxy for biomass. By systematically switching the tagged isolate across experiments, all pairwise interactions between strains can be mapped. (b) Multi-fluorescent labelling approach: Microbial species are labelled with distinct fluorescent proteins, allowing their identification. They are randomly co-encapsulated, incubated, and monitored over time. Growth trajectories for each initial combination reveal single-cell interaction dynamics. (c) Focal species community screening: A single fluorescently tagged focal species is randomly co-encapsulated with community-derived microbial cells. After incubation, droplets are screened based on focal species fluorescence intensity, measuring single-cell interaction effects on the focal species enabling downstream sorting and sequencing to associate droplet compositions with focal species growth outcomes. (d) Random co-encapsulation: Community members are randomly encapsulated. Post-incubation, droplets are sorted based on overall biomass or functionality. Sequencing identifies the microbial consortia associated with specific endpoint phenotypes.

To explore interactions using cultured isolates, two approaches are generally used. (i) In a first approach, a labelled strain of focus (focal strain) is paired with each of the other strains in the synthetic community (Fig. 3a). Strain identity is inferred from the label for the focal strain (i.e. a genetically encoded fluorescent protein), droplet markers are used to infer the identity of the other strains. Droplets are made for all strains and then randomly paired and fused. The biomass of the focal strain is tracked and interactions are derived using the droplet markers (i.e. identity of partner strain is derived yielding the investigated interaction). This process is done for all strains of focus, yielding high-throughput co-cultures of pairwise and higher order interactions.

This method was first described by (Kehe et al. 2019). In their pioneering work, they introduce a chip (“kChip”) that allows random assembly of 100 000 s synthetic communities in a single day. From a collection of n species, n droplet populations are made, after which they are mixed, and loaded on the chip. The kChip contains structured microwells that groups the droplets into clusters of k droplets each. After the chip is loaded, the droplets in the clusters are fused and incubated. The microwells fix the position of the droplet, allowing readdressing of the same droplet over time. Using this method the authors were able to study the influence of 19 partners and 6 carbon sources on the yield of focal species *Herbaspirillum frisingense* (a model plant symbiont). From their screen they found microbial consortia that robustly promote symbiont growth over all carbon sources, indicating that certain microbial consortia maintain functional traits despite variations in environmental factors.

Several notable high-throughput studies have since made use of the kChip. In a follow-up study, Kehe et al. (2021) measured over 180 000 interactions among 20 soil bacteria across 40 carbon environments. They found interactions where at least one of the partners was positively affected by the other, such as parasitism and commensalism, were common, occurring in about 40% of cases. In addition, they found that in 85% of cases, non-growing strains were promoted by strongly growing strains. Baichman-Kass et al. 2023 analyzed over 14 000 bacterial communities composed of combinations of 61 soil and leaf isolates, measuring their effects on six focal species. They found that negative interactions were highly non-additive, with the strongest single-species effect dominating the joint outcome in 77.1% of species pairs. A third study by Gopalakrishnappa et al. (2024), focused mainly on permutating environmental conditions and their effect on algae-bacteria interactions. They analyzed over 100 000 microbial communities across 525 environmental conditions. Their findings show that pH and buffering capacity substantially alter algae-bacteria interactions by affecting the impact of nutrient levels on growth.

Overall, kChip workflows enable the rapid elucidation of design principles for synthetic microbial communities. While similar studies have been conducted using microtiter plates (Wittebolle et al. 2009, Bai et al. 2015, Carlström et al. 2019), these methods are significantly more labor-intensive, requiring extensive pipetting and handling. The simple manipulations and high-throughput nature of the kChip platform make it a highly valuable tool for exhaustively characterizing microbial interactions on labeled strains. However, it is important to note that complex environments—such as plant inoculation systems (e.g. Carlström et al. 2019, Y.-W. He et al. 2008)—are not easily represented in a droplet format. In such cases, large-scale experiments tailored to the specific environment are a favored approach. Another limitation of the kChip is that it cannot determine single-cell interactions (see Box 1) since it starts with multi-cell inoculated droplet populations and thus determines the interactions on a population level.

(ii) A second approach consists of directly co-inoculating the desired synthetic community in a single droplet population. Individual microbes are randomly encapsulated and identified using different fluorescent labels for each species (Fig. 3b). This approach is relatively simple, but it is limited by the number of fluorescent labels available and the molecular tools available for engineering the desired strains. The most notable study using this approach is the 2019 paper “Microbial Interaction Network Inference in microdroplets” (Hsu et al. 2019). This method randomly co-encapsulates labelled microbes and monitors them using their fluorescent signal. Advanced image analysis then allowed the quantification of pairwise and higher-order single-cell interactions. The many replicates and single-cell level encapsulation allowed measuring and modelling variable nature of single-cell microbial interactions.

#### Native interaction profiling: interaction screening using extracted native communities

In contrast to approaches using cultivated isolates, native interaction profiling works with natural microbial communities, without isolating individual members. In these experiments, microbes are extracted from their environment and used directly for encapsulation, preserving the original richness, and the presence of both active and dormant cells. This makes complex community screens ecologically informative, capturing aspects of natural community function that are difficult to reconstruct from isolated strains.

Droplet microfluidics, at first glance, offers an elegant route for screening complex communities: it avoids prior cultivation and enables random pairing of microbes at ultrahigh throughput. However, avoiding cultivation adds considerable complexity. Strain identities are unknown a priori, and labeling techniques to infer strain-level identity are often incompatible with high-throughput biomass measurements (Kuchina et al. 2021, Gaisser et al. 2024).

Despite these challenges, complex community interaction screens have been developed, although efforts so far remain limited. One successful strategy pairs a complex community with a labeled, cultivable focal strain (e.g. tagged with a fluorescent protein; Fig. 3c). This setup allows the single-cell interaction effects of millions of individual cells on the focal strain to be rapidly assessed (Terekhov et al. 2017, 2018, 2020, Mahler et al. 2021, Baranova et al. 2022). The typical aim of such screens is to identify community members that either promote or inhibit the growth of the focal strain, providing insights into beneficial or antagonistic interactions from the extracted community with the focal strain. In addition, random partnering cocultivation approaches have been explored, where community members are randomly encapsulated and grown together in droplets (Man et al. 2024, Fig. 3d). However, the initial composition of droplets is typically unknown until after droplet sorting and sequencing, limiting the information such experiments bring. Overall, while these workflows demonstrate the potential of droplet microfluidics for studying complex communities, further methodological developments are needed to fully realize their promise. We discuss the insights gained from current studies (see: Focal species interaction screening reveals rare functionalities) and future directions (see: Interaction screening) in later sections.

## Lessons learned

With a growing set of high-throughput tools available, we now turn to the insights they have enabled. Focusing on single-cell (or single-cell outgrowth) physiology, this section highlights key discoveries in microbial ecology that have emerged from applying these workflows.

### Interactions are variable at the single-cell level

Traditionally, microbial interactions are assessed by comparing growth in monoculture and co-culture within well-mixed, microliter-scale liquid environments, which generally yield consistent and predictable outcomes. However, these bulk assessments do not take into account the role of single-cell heterogeneity in interaction outcomes.

By seeding droplets with cells of the interacting microbial species (Fig. 2), recent studies have demonstrated that interaction outcomes show important variability on a single-cell level. Batsch et al. (2024) explored microbial interactions by encapsulating microbial cells in picoliter droplets (1–3 cells per droplet). They found that single-cell interaction assay outcomes—ranging from substrate competition to antagonism and cell killing—were more variable in droplets than in bulk cultures. In some cases, outcomes were even reversed; for instance, strains that were typically outcompeted in uniform conditions were able to thrive or dominate in droplets. This variability was attributed to both stochastic assembly of founder populations and inherent phenotypic differences among individual cells, with the effect most pronounced at low starting densities and diminishing as the number of founder cells increased. A report by Hsu et al. (2019) used a similar approach to show that average interaction outcomes in droplets were consistent with macroscale cultures but also reported a similarly broad interaction distribution, suggesting stochastic effects attributing to variability of interaction outcomes. Finally, Guo et al. (2019) observed similar patterns in microdroplet cocultures of *E. coli* and *Enterobacter cloacae*, reinforcing that founder cell heterogeneity can lead to a spectrum of outcomes, from competition to mutualism, even under consistent conditions. In addition to phenotypic variability, they proposed that bacterial sensing and perception of neighboring species may further shape interaction dynamics. These findings underscore that microbial interactions are not static properties but are highly variable on a single-cell level and are influenced by spatial structure, environmental and community context and phenotypical heterogeneity (Batsch et al. 2024).

### Physiological phenomena are variable at the single-cell level

Beyond multi-species interactions, droplet-based single-cell outgrowth studies of clonal populations have consistently revealed variability in core physiological processes, including growth, stress survival, antibiotic resistance, quorum sensing, and phage susceptibility. These observations highlight that even clonal populations harbor significant functional heterogeneity.

Studies by Barizien et al. (2019) and Taylor et al. (2022) demonstrated that growth dynamics in *E. coli* are inherently stochastic: individual cells divide independently and exhibit variable division times, consistent with predictions of the Bellman-Harris model. This variability extends to stress responses, as shown by Pratt et al. (2019), who found that deletion of the hpf gene in *Pseudomonas aeruginosa* PA01–responsible for ribosomal inactivation during starvation –led to a broader distribution of lag phases following starvation.

Droplet workflows have also been instrumental in uncovering heterogeneity in antibiotic responses. Lyu et al. (2018) developed a microfluidic method to phenotype antibiotic heteroresistance—a phenomenon where a microbial population contains both susceptible and resistant subpopulations—from single-cell outgrowth, using encapsulation of individual *E. coli* cells in droplets and fluorescence-based detection of viability after antibiotic exposure. Their experiments were highly sensitive, detecting subpopulations as small as 10^−6^ of the entire bacterial population. Additionally, they tracked the emergence of heteroresistance after exposing cells to sub-lethal antibiotic doses, observing a gradual increase in resistance frequency over several days. Another study explored the distribution of minimum inhibitory concentration across a monoclonal population and found significant variability in growth inhibition across individual cells (Scheler et al. 2020).

Quorum sensing, often thought of as a coordinated, population-level behavior, also displays unexpected variability at the single-cell level. In their study, Boedicker et al. (2009) explored quorum sensing in *P. aeruginosa* of single-cell or multi-cell outgrowth. In one experiment they looked at the expression behavior of quorum sensing-dependent gene *lasB* fused to a green fluorescent protein. They showed that small groups of bacteria, as few as one to three cells, could initiate quorum sensing with a marked heterogeneity, where only 20% of droplets with single cells activated quorum sensing within 10 hours. In a second experiment, the researchers demonstrated that quorum sensing activation was essential for growth in media with adenosine as the sole carbon source. Adenosine catabolism is regulated by quorum sensing and will only occur after a threshold value is reached. Here they saw that 2 out of 14 droplets with one to three cells initiated quorum sensing and supported bacterial growth after 29 hours, further highlighting variability in quorum sensing -dependent behavior.

Finally, Nikolic et al. 2023 combined droplet microfluidics with time-lapse fluorescence microscopy to study *E. coli* population dynamics during phage infection at high temporal resolution. Their work confirmed that different phages induce distinct lysis patterns—DNA phage T7 caused rapid and near-complete lysis, while RNA phages MS2 and Qβ produced slower and more variable outcomes. Critically, they also uncovered substantial single-cell outgrowth heterogeneity within clonal bacterial populations: even under the same phage challenge, individual cells showed divergent fates, including delayed lysis, continued growth, or survival. This variability highlights the importance of unknown deterministic and stochastic processes in bacteria-phage interactions and demonstrates how droplet-based approaches can reveal hidden functional diversity critical for understanding eco-evolutionary dynamics.

### Droplet isolation tends to improve recovery of diversity

Droplet-based culturing is often cited as a potential solution to the great plate count anomaly (Z. He et al. 2022, Kaminski et al. 2016, Staley and Konopka 1985). Here we investigate whether growing bacteria in a droplet offers superior isolation capabilities (i.e. a larger percentage of unique species of the original sample will grow) over traditional methods by (i) evaluating possible mechanisms and (ii) reviewing empirical results in literature.

Bacterial uncultivability is thought to be driven by a variety of factors: unknown nutritional needs, competitive overgrowth of faster-growing species in standard cultivation practices, dormancy states, obligate microbial partnerships, and quorum sensing-dependent growth triggers (Stewart 2012, Epstein 2013, Lewis et al. 2021). In cases where unknown nutritional needs limit cultivability, neither droplet-based nor plate-based methods provide a solution as the needs remain unknown. Similarly, techniques like dilution-to-extinction can effectively reduce the influence of competition without the need for droplets (Song et al. 2009).

However, droplet-based culturing could present distinct advantages for overcoming dormancy-related issues and facilitating quorum sensing-dependent growth triggers. By allowing the testing of millions of individual dormant or non-dormant cells, droplet microfluidics increases the likelihood of discovering active strains. As mentioned before, its ultrahigh throughput also allows efficient random pairing of bacteria, therefore increasing the chance that an obligate symbiotic partnership is found. The method's ability to isolate individual cells in minute volumes, equating to high effective concentrations (1 cell per 10 pL droplet is equivalent to 1 × 10^8^ cell/mL), starkly contrasts with the lower concentrations achieved in dilution-to-extinction approaches (1 cell in 10 μL is equivalent to 10^2^ cell/mL). This concentration benefit can significantly enhance the activation of growth in cases where it is quorum-sensing-dependent (Boedicker et al. 2009, Striednig and Hilbi 2022) and could also better allow the study of obligate partnerships by ensuring close micrometer scale cellular interaction (Co et al. 2020). However, a high cell concentration could just as well inhibit growth in quorum sensing-dependent processes or halt growth by rapid accumulation of waste products (Striednig and Hilbi 2022).

Empirical results generally show that droplet-based cultivation recovers a larger fraction of the original microbial richness compared to traditional techniques such as plate culturing. In their 2020 paper, Watterson et al. compare plate-based with droplet-based culturing in their efficacy to isolate members from a human stool sample. They find that while richness (i.e. number of detected ASVs) was significantly larger in droplets—increased between 15% and 410% depending on growth medium—Shannon diversity was not (Watterson et al. 2020). A paper by Mahler et al. (2021) made the same comparison for a soil community (albeit using a single growth medium) and found that both total number of OTUs and Shannon diversity was significantly higher in droplet-based than in plate-based culturing. McCully et al. (2023) grew a stool sample using droplet-based culturing and compared the grown community to the community obtained after growing in a batch incubation. Here, no significant difference was found between the richness of both culturing methods, however the unique identities of the enriched ASVs were found to be significantly different. *Phascolarctobacterium faecium* was also enriched which is notably difficult to isolate (Fodor et al. 2012). In a recent study, Man et al. (2024) enriched the candidate phyla radiation (CPR)—a hard-to-culture monophyletic clade—up to 13-fold using droplet cultivation, compared to batch incubation, which only enriched CPR members up to 2-fold.

Although the observed increased richness in isolated taxa could be solely due to increased throughput allowing better sampling of active members, there are potentially other mechanisms at play. In particular, droplet-specific conditions could be permissive to growth and could foster *in situ*-like conditions or enable ‘soft’ interactions. This would involve mechanisms such as gas exchange between droplets (e.g. H_2_ as an electron donor (McCully et al. 2023)), exchange of dissolved molecules between droplets through cross-talk—a process of which the importance is poorly understood for biological experiments and could enable aforementioned soft interactions (exchange of molecules between two droplets containing bacteria) (Etienne et al. 2018, Waeterschoot et al. 2024) –, or quorum sensing-dependent effects (Boedicker et al. 2009). One piece of evidence supporting that droplet context is permissive to growth, is the difficulty in isolating bacteria from droplets, with repeated reports failing to scale up droplet-grown bacteria to microliter-scale cultures (Mahler et al. 2021, McCully et al. 2023, Man et al.^2024^). This consistent failure to scale up suggests that growth is coupled to droplet-specific conditions, which may create a unique microenvironment that cannot be readily replicated in traditional culture formats.

### Focal species interaction screening reveals rare functionalities

Droplet microfluidics enables direct screening of communities at the single-cell level, bypassing the biases of traditional isolation methods and preserving the phenotypic states of microbes as they exist in their natural communities. As seen previously, this native interaction profiling could be a powerful capability for microbial ecology, allowing interaction profiling without prior culturing. Several studies have leveraged this approach by co-inoculating microbiome members with a fluorescently labeled focal species (Fig. 3c), enabling high-throughput identification of interacting strains.

One notable example is the study by Terekhov et al. (2018) who performed a single-cell interaction assay between a complex microbial community and a focal strain, *Staphylococcus aureus*. By co-inoculating individual members of the oral microbiota of Siberian bears with *S. aureus* in microdroplets and employing FACS, they identified strains that inhibited the growth of the focal strain. This approach led to the discovery of a *Bacillus pumilus* strain producing amicoumacin A, an antibiotic effective against *S. aureus*. In a follow-up study, microbial communities of various wild animals were screened leading to the repeated isolation of *B. pumilus* strains producing amicoumacin A (Baranova et al. 2022). Mahler et al. (2021) used a similar rationale to identify soil members that inhibit growth of focal species *B. subtilis* and *E. coli* and found a *Bacillus* sp. that inhibited both species. Growth-boosting species interactions have also been detected in this way. In a 2019 study, Ohan et al. screened freshwater microbial communities for microbes that promoted the growth of the algae *Chlorella sorokiniana* and found that *Pseudomonas mendocina, Variovorax paradoxus* and *Ralstonia pickettii* could be found in high-biomass droplets (Ohan et al. 2019). Isolation of *Pseudomonas* spp. showed that in co-culture with the algae, the algae had a > 30% improvement in growth rate compared to monoculture.

While these studies show the capability of microfluidic screening to discover rare isolates with strong functional effects, they stop short of characterizing the interaction spectrum of those isolates (i.e. the magnitude and frequency of positive and negative interactions). Additionally, the ecological relevance of the focal strain in the context of the screened microbiome is not reported. An approach that would be more ecologically informative, would involve screening focal strains, isolated from their native microbiome, against their native microbiome community to map their full interaction profiles. This could illuminate the functional niches of both cultured and uncultured members, deepening our understanding of microbial community structure and dynamics.

## What lies ahead

In previous sections, we have shown the capability of droplet microfluidics to deal with the diversity encountered in microbiomes. It functions as a platform, allowing custom high-throughput assay development, easily dealing with the single-cell level diversity encountered in microbiomes. We have seen that even with a limited number of applications, new knowledge and insights have been generated. Notably, it has revealed that microbial behavior at the single-cell level is highly variable—ranging from variable growth dynamics and antibiotic resistance to heterogeneous responses to viral infection and quorum sensing. These findings have broad implications for interpreting microbiome processes such as colonization, spatial organization, and functionality.

In this section, we further explore the potential of droplet microfluidics to assess single-cell functional states and interactions across full microbiomes. The discussion is framed as a forward-looking perspective, informed by current knowledge and capabilities. The goal is to illustrate how these technologies could push the boundaries of single-cell microbiome research. We conclude by addressing fundamental limitations, caveats and biases inherent to droplet workflows and highlight potential advancements or solutions to mitigate these.

### High-throughput experimentation

With relatively limited applications so far, droplet microfluidics has already offered valuable insights into microbial life at the single-cell level. However, most applications are relatively simple, although the technology lends itself to sophisticated workflows due to its modular character (Moragues et al. 2023). With further development, such as advanced droplet tracking and integration of selective droplet splitting, fusion and sorting, one could envision droplet workflows as billion-well plates, with droplets as programmable, picoliter-scale microtiter wells. Subpopulations with desired phenotypes could be selectively extracted and split, after which the split daughter droplets could be subjected to different analyses such as single-cell -omics (Z. Li et al. 2019, Y. Liu et al. 2022, H. Li et al. 2024). Such capabilities would support targeted, iterative experiments advancing our ability to explore microbial behavior at microbiome-scale and create understanding by hypothesis-based testing.

We also note that in this context, droplet microfluidics offers practical opportunities to advance the study of microbial uncultivability. The ability to rapidly isolate and experimentally test large numbers of individual cells under a wide range of defined conditions allows for more exhaustive sampling of microbial communities, including the rare or slow-growing members that are typically overlooked. Such approaches can help identify the environmental, nutritional, or ecological factors required for growth. However, to fully understand which bacteria fail to grow and why, droplet-based experiments should be combined with complementary methods such as traditional plating and dilution-to-extinction, single-cell RNA sequencing or FISH-based taxonomic labeling to link growth outcomes to microbial identity, expression and physiology. Together, these technologies provide a framework to experimentally address the long-standing question of the dominating mechanisms behind why many bacteria remain uncultivable.

### Microbiome-scale functionality screening

Single-cell heterogeneity in microbiomes can take various forms. Of particular ecological significance is the emergence of functional guilds within genetically similar or clonal populations—where cells adopt distinct roles, enabling resource partitioning or metabolic cooperation. For example, even in the simplest version of a microbiome—a pure culture—a clonal population of *B. subtilis* has been found to divide into two distinct interacting metabolic subpopulations, namely one that produces acetate and another that consumes acetate producing acetoin (A. Z. Rosenthal et al. 2018). The authors proposed that this represents an ecological strategy in which the strain modulates its environment via functional state-switching within a clonal population. Another study showed that clonal populations of *E. coli* exhibited cell-to-cell heterogeneity when exposed to a glucose concentration gradient on a 2D-surface (Co et al. 2019). Cells differed in growth rates and expression levels and evidence for metabolic cross-feeding between glucose-fermenting and acetate-respiring subpopulations was found. At the community level, it has been shown that a single species of *Bacillus succiniproducens* fills in different metabolic niches, driven by biofilm formation pathway genes in the rumen microbiome (Jia et al. 2024).

It is clear that this kind of functional heterogeneity is a fine-grained driver of community structure and functionality in microbiomes and measuring it would lead to better understanding of a microbiome state. However, this kind of heterogeneity is hard to profile using traditional techniques. Firstly, (classic) -omics are bulk analyses that pool sequences from many microbiome members and make individual-level interpretations difficult. A more cutting edge approach is single-cell transcriptomics, which has proven very insightful for eukaryotic studies over the past two decades and has recently seen exciting developments for the study of prokaryotic organisms (Kuchina et al. 2021 B. Wang et al. 2023, Jia et al. 2024). However, while transcriptomics offer crucial expression-level insights in microbiomes, they measure phenomena several levels removed from the resulting microbial functionality, limiting interpretability of such data (Buccitelli and Selbach 2020). To understand what a microbial cell is actually doing in a microbiome—how fast it is growing, what substrate it is consuming, whether it is competing, cooperating, or persisting in dormancy—we must grow it and probe its functionality in a relevant context. Isolation is the gold standard method to grow microbial cells and study their physiology and resulting functionality. However, it does not paint a single-cell picture of microbiomes since it is practically not feasible to isolate at the level of the single cell.

It is clear that to enable better understanding of the heterogeneity of functionality at a microbiome level, a methodological niche needs to be filled. Droplet microfluidics could fill this niche due to its high throughput, its high level of control and the direct linking of genotype and functionality. To assess the diversity of functions, droplet-based single-cell functionality screens can be performed to ‘fingerprint’ the state of a function in a microbiome and assign taxonomy to the different functional groups.

**Figure 4.**
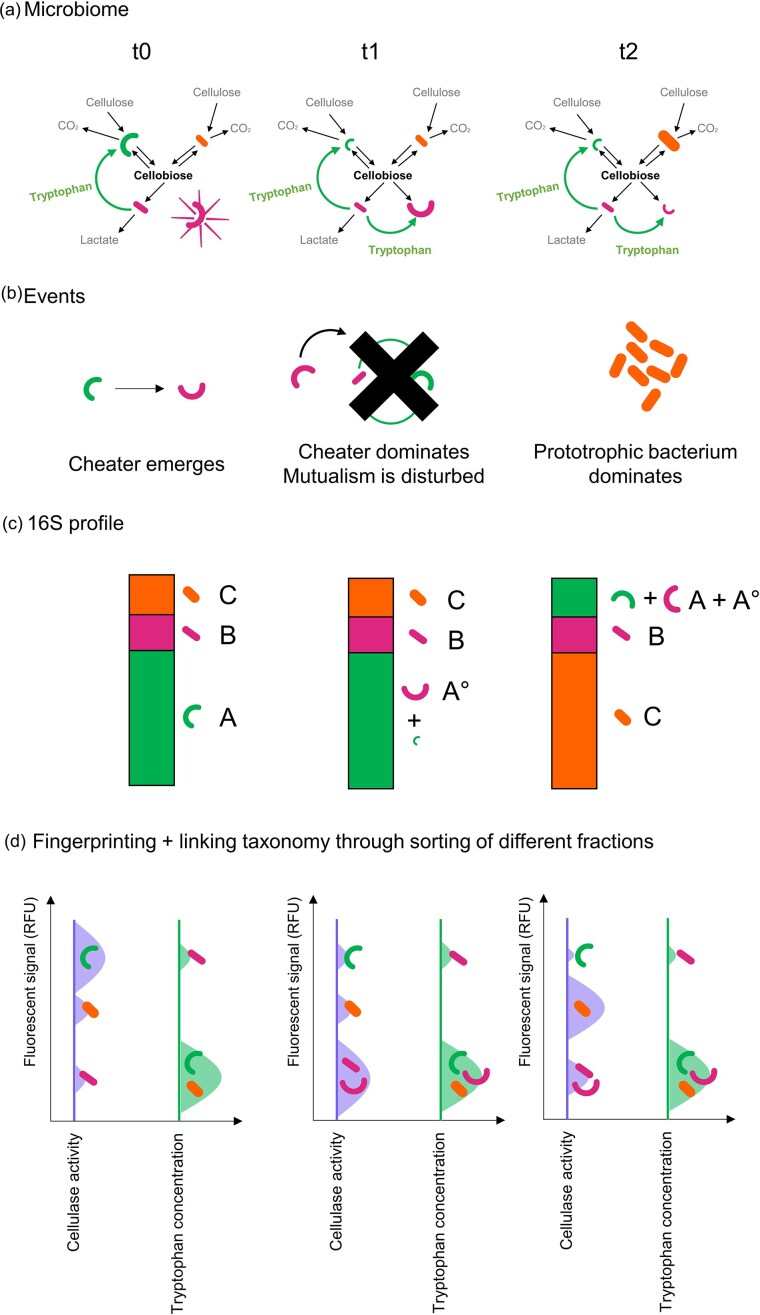
Dissecting functional dynamics in a simple cellulose-degrading community using droplet-based single-cell screening. (a) Time-resolved schematic of a cellulose-degrading community composed of four members: the mutualistic pair A (green curved rod) and B (magenta bacillus), a prototrophic degrader C (orange bacillus), and the emergent non-cellulase-producing mutant A° (magenta curved rod). (b) Key ecological events driving system dynamics, including the emergence of A° at t0, its takeover at t1, and the collapse of mutualism leading to the rise of C at t2. (c) 16S rRNA-based taxonomic profiles over time illustrate shifts in community composition but fail to resolve functional changes such as the rise of A°, as it is indistinguishable from A in standard taxonomic data. (d) Density distribution of flow cytometry measurements of double emulsions reporting on cellulase activity (left) and tryptophan secretions (right). The y-axis reflects the fluorescence signal corresponding to the functional readout and the x-axis shows the relative amount of droplets that show that level of fluorescence. This enables detection of functional guilds and the emergence of A°, which are invisible to bulk approaches. Functional clusters are sorted and sequenced, linking phenotype to genotype.

As an example, consider a cellulose-degrading microbiome, in a carbon-limited environment (Fig. 4a). There are three species of bacteria present: a fast-growing, tryptophan-auxotrophic cellulose-degrader (A) which secretes cellulases to produce cellobiose extracellularly, a cellobiose-consuming bacterium which secretes tryptophan (B), and a slow-growing, prototrophic cellulose-degrader, also secreting cellulases to produce cellobiose extracellularly (C). Bacteria A and B are in an obligate mutualism, exchanging cellobiose and tryptophan.

At t0 a mutant bacteria A° is formed that does not secrete cellulase, allowing it to grow faster and profit off common goods cellobiose and tryptophan. At t1 A° takes over the community and becomes the majority strain of species A. Since both A and A° are now competing for tryptophan and since A° is competing with B for cellobiose without also producing it extracellularly. At t2, a less efficient partnership is formed, causing a decline in relative abundance of species A and B, with C taking over the community since it experiences less competition for cellulose from species A (Fig. 4b). Screening this community using single-cell measurements of cellulase activity and tryptophan concentration in droplets shows their evolution over time and allows elucidating driving events in the microbiome. Fingerprint information (i.e. the distribution of a single-cell functionality assay signal) reveals the different functional clusters. It contains information on how active they are, how numerous they are, and—after sorting and sequencing—who they are. In this case functionality screening captures the persistent existence of tryptophan secretors, and at t1, the decrease of highly active cellulase producers A together with the emergence of a large non-cellulase producing population A°, allowing fine-grained understanding of the microbiome.

If this example were to be studied with bulk omics, functional signals would be averaged across the entire community, obscuring heterogeneity and masking the emergence of subpopulations like the non-cellulase-producing mutant A°. As a result, key dynamics—such as the breakdown of mutualism or the selective advantage of cheaters—would likely go undetected, limiting insight into the functional drivers of microbiome change (Fig.4c).

In practical terms, a workflow like this would mean extracting the cells from their environment, encapsulating the cells at a single-cell level in droplets together with a fluorescent cellulase activity indicator and a fluorescent tryptophan indicator. After incubating the cells in this assay medium the droplets can be measured and sorted. Measuring the droplets leads to the pictured distributions (Fig. 4d). Sorting the droplets based on assay signal followed by sequencing allows appointing a 16S identity to each distinct signal distribution, which allows extracting the heterogeneous ecological role of each bacterial species. Sorting of droplets into wells with media and growing them into lab-scale cultures allows quick and targeted extraction of bacteria of interest, aiding isolation efforts.

### Interaction screening

Droplet-based interaction screening has several characteristics that makes it an ideal candidate for future interaction screening workflows. Firstly, it combats combinatorial explosion associated with interaction screening to a certain extent, allowing feasible generation and testing of maximum up to 10^10^ droplets—for 10 pL droplets, with an upper droplet generation and analysis speed in the order of magnitude of 10 000 droplets/sec, each step would take 11.57 days, setting the upper limit of feasibility. Secondly, it allows exploring the single-cell level variability of interactions, which is impossible with bulk analysis (Batsch et al. 2024) Exploring different interaction outcomes and understanding the reasons behind them leads to a better understanding of microbial community assembly.

For screening single-cell interactions of synthetic communities with fluorescently labelled isolates, current workflows can be expanded by making use of upcoming spectral technologies (Hsu et al. 2019, K. Chen et al. 2021, Konecny et al. 2024). Spectral flow cytometry and spectral microscopy are similar to their ‘normal’ fluorescent counterparts but have many more emission detectors which they use to capture the full spectrum of each fluorophore, after which unmixing algorithms are used to distinguish the signal coming from each fluorophore. The multitude of detectors—currently up to 186 in ID7000 (Sony)—allows for the simultaneous use of more fluorescent molecules at once, with panels of up to 50 different colors having been designed (Sharma et al. 2024, Konecny et al. 2024). Similarly, such an expanded panel of tagged microbes, each labeled with its own fluorescent protein, could lead to straightforward high-throughput interaction testing in droplets. Random recombination during encapsulation (Fig. 2) at high throughput, combined with dynamic monitoring using spectral microscopy could resolve single-cell interaction patterns of large libraries of labelled strains quickly and with minimal hands-on time. However, a major bottleneck remains in the reliable chromosomal labeling of non-model microbes with distinct fluorescent proteins. In our view, this challenge has principally been responsible for the limited application of high-throughput, fluorescence-based interaction testing across diverse communities, highlighting a key area for future methodological development.

Direct community interaction screening—i.e. elucidating interactions between members from a microbiome without relying on isolation—is a more complex endeavor. Since fluorescent protein labelling cannot be used without isolation, tracking the identity of strains contained within a droplet needs to be done using other tools. Through timeseries image analysis, founder cells in a droplet can be tracked over time to infer strain identity and show population dynamics within each droplet (Todorov et al. 2023). After incubation, location-specific barcoded 16S primers with PCR mix can be injected in droplets after which they are collected off-chip. A heat lysis step and a PCR reaction are sufficient for library preparation and subsequent sequencing of barcoded 16S sequences which in its turn allows assigning taxonomy to the location-specific observed droplet data. Interactions are then inferred by comparing to monoculture data, which is generated using the same approach.

Direct community interaction screening would allow high-throughput testing of interaction hypotheses brought forward by microbial network inference. Given the dearth of fully resolved microbial interaction networks, such interactions will also be useful as benchmark data to assess the accuracy of microbial network inference algorithms (Faust and Raes 2012, Faust 2021).

## Limitations, caveats and biases

Droplet microfluidics is a promising platform for advancing single-cell microbiome research, due to its high throughput, fine control over individual cells, and assay flexibility. However, despite these advantages, currently droplet applications still see limited use in microbiological applications. This is mostly due to its technical complexity, since most microfluidic unit operations require custom microfluidic analysis stations (B. L. Wang et al. 2014, Panwar et al. 2023). Work has been done to reduce the complexity of droplet workflows, with droplet generation and double emulsion generation (for FACS compatibility) able to be done on standard lab equipment (Shin et al. 2019, J. Wang et al. 2022). However, for more complex unit operations, such as addition of liquids or removal of liquid to or from droplets, work is still required to enable widespread use, either through commercialization of microfluidic chips and microfluidic analysis stations (Breukers et al. 2025) or through simplification and open-source sharing of protocols, equipment designs and chip design (Wenzel 2023). An additional major limiting factor is that the droplets’ small size does not allow straightforward measuring of any target functionality, molecule or phenomenon since they have to be translated to a measurable assay. The need for (mostly) custom development of assays for each specific functionality adds complexity and resource demands.

Another fundamental limitation of droplet microfluidics is the Poisson-based encapsulation process (see section “Droplet generation and encapsulation of bacteria”). While single-cell droplets dominate at low lambda values, increasing lambda leads to droplet populations containing a mix of founder cell numbers (i.e. multi-cell inoculation), making it essential to determine the initial composition of each droplet when analyzing interactions or functions. This is particularly relevant when studying complex communities, where limited labelling strategies are available. Current strategies to identify initial conditions include microscopy, fluorescent labeling, or sorting followed by 16S rRNA sequencing. However, each approach involves trade-offs: microscopy limits throughput, while techniques like FACS preclude dynamic, time-resolved measurements, highlighting the technological challenge of accurately capturing initial droplet states without compromising experimental resolution or scale. Future developments could mitigate such concerns by making use of molecular techniques such as barcoded beads which are already widely used in single-cell RNA sequencing. Another approach to combat the limits of random Poisson loading is to move to more deterministic loading—where the number of cells per droplet is known and controlled. For this, several strategies have been developed. These include post-generation droplet sorting, inertial ordering of cells before encapsulation, and on-demand encapsulation (reviewed in Collins et al. 2015).

Another practical limitation comes in the form of droplet cross-talk (i.e. the migration of compounds from one droplet to another). Droplets offer effective physical isolation of individual cells, however, chemical isolation is more nuanced. The surfactant-stabilized droplet interface forms a mechanical barrier that prevents cell exchange, but it does not fully block molecular transport. In many cases, especially for hydrophilic compounds, cross-talk is minimal or negligible—for example, auxotrophic strains behave as expected in nutrient-limited screens, showing that the used amino acids remain well-confined (Hsu et al. 2019, Tan et al. 2022). However, this cannot be assumed for all compounds. Molecules with high hydrophobicity, such as caffeine, can diffuse through the continuous oil phase or be transported via excess surfactant not bound at the droplet interface (Payne et al. 2023). Such phenomena become particularly problematic when using complex media, where the composition is not well-defined and it becomes difficult to predict which molecules might leak between droplets. Although the overall risk for cross-talk may be low in many experimental setups, it remains poorly characterized and likely underestimated. Strategies such as lowering surfactant concentration (Kulesa et al. 2018), using dendronized surfactants (Chowdhury et al. 2019), nanoparticles (Pan et al. 2014, Waeterschoot et al. 2024), and optimizing oil phase composition (e.g. perfluorinated oils) can help reduce unintended transfer (Payne et al. 2023). Still, a more systematic understanding of molecule-specific transport and droplet boundary behavior is needed to ensure robust experimental design (Etienne et al. 2018).

In the context of ecological knowledge generation, the confinement of individual microbial cells or consortia within microdroplets inherently leads to environmental context omission. Microbial cells in their natural settings experience complex and dynamic gradients of nutrients, oxygen, pH, and signaling molecules, along with interactions with a diverse array of other organisms and abiotic surfaces. The environment within a microdroplet strips away these environmental cues that can significantly influence microbial behavior. Similarly, community context omission arises from the isolation of single cells or small groups within droplets. Natural microbiomes are characterized by intricate interspecies interactions, including competition, cooperation, and communication via quorum sensing. By compartmentalizing cells, droplet microfluidics disrupts these complex network dynamics, potentially altering individual cell behavior and precluding the study of emergent community-level properties that arise from these interactions. While this isolation allows for the focused study of individual entities, it's crucial to acknowledge that the insights gained may not fully reflect the ecological realities of the microbes within their native communities.

## Conclusion

Single-cell approaches are becoming increasingly important in microbial ecology, offering a level of resolution that bulk methods cannot achieve. By observing variation at the level of individual cells, we can access ecological patterns and functional behaviors that are often obscured when averaging across populations. This is particularly relevant in microbiology, where species definition is particularly fluid and taxonomy alone does not reliably predict function. In this context, the individual microbial cell can become a key unit of analysis, and methods that enable controlled, high-resolution experimentation are essential for building a more accurate understanding of microbial communities.

As reviewed here, droplet microfluidics provides a flexible and powerful platform for investigating microbial physiology and function at a microbiome scale. In its current form, it has revealed important phenomena, such as variable growth behaviors, the heterogeneous nature of interactions, and rare functional states. However, accessibility remains a key limiting factor: compared to more mature and standardized technologies, droplet-based approaches often require custom assay development and specialized expertise, which restricts widespread adoption. Moving forward, improving ease of use—either through standardization, commercialization or broader dissemination of existing workflows—will be essential for droplet microfluidics to realize its full potential. While it is not a complete solution on its own, it complements other single-cell tools and offers a promising experimental framework to better link microbial identity to function, helping push microbial ecology toward a more mechanistic and predictive field.
